# 2-Amino­pyrimidin-1-ium 4-methyl­benzene­sulfonate

**DOI:** 10.1107/S1600536811018198

**Published:** 2011-05-20

**Authors:** Masoumeh Tabatabaee, Najmeh Noozari

**Affiliations:** a Department of Chemistry, Yazd Branch, IslamicAzad University, Yazd, Iran.

## Abstract

In the crystal structure of the title compound, C_4_H_6_N_3_^+^·C_7_H_7_O_3_S^−^, inter­molecular N—H⋯O hydrogen bonds link the cations and anions into chains along [100]. Additional stabilization is provided by weak C—H⋯O hydrogen bonds. An inter­molecular π–π stacking inter­action with a centroid–centroid distance of 3.6957 (7) Å is also observed. The H atoms of the methyl group were refined as disordered over two sets of sites with equal occupancies

## Related literature

For related structures, see: Tabatabaee *et al.* (2010 ▶, 2011 ▶).
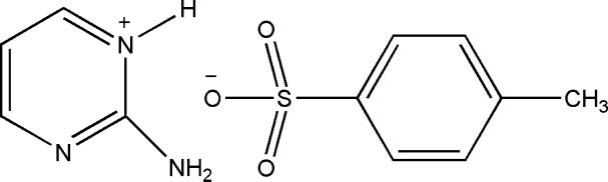


## Experimental

### 

#### Crystal data

C_4_H_6_N_3_^+^·C_7_H_7_O_3_S^−^
*M* *_r_* = 267.30
Monoclinic, 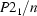

*a* = 6.2567 (3) Å
*b* = 13.3756 (6) Å
*c* = 15.2512 (7) Å
β = 101.335 (1)°
*V* = 1251.43 (10) Å^3^
*Z* = 4
Mo *K*α radiation
μ = 0.26 mm^−1^
*T* = 100 K
0.50 × 0.36 × 0.32 mm

#### Data collection

Bruker SMART APEXII diffractometer
12275 measured reflections
3617 independent reflections
3160 reflections with *I* > 2σ(*I*)
*R*_int_ = 0.021

#### Refinement

*R*[*F* ^2^ > 2σ(*F* ^2^)] = 0.032
*wR*(*F* ^2^) = 0.088
*S* = 1.04
3617 reflections
163 parameters
H-atom parameters constrained
Δρ_max_ = 0.45 e Å^−3^
Δρ_min_ = −0.38 e Å^−3^

### 

Data collection: *APEX2* (Bruker, 2005 ▶); cell refinement: *SAINT* (Bruker, 2005 ▶); data reduction: *SAINT*; program(s) used to solve structure: *SHELXTL* (Sheldrick, 2008 ▶); program(s) used to refine structure: *SHELXTL*; molecular graphics: *PLATON* (Spek, 2009 ▶); software used to prepare material for publication: *SHELXTL*.

## Supplementary Material

Crystal structure: contains datablocks I, global. DOI: 10.1107/S1600536811018198/lh5246sup1.cif

Structure factors: contains datablocks I. DOI: 10.1107/S1600536811018198/lh5246Isup2.hkl

Supplementary material file. DOI: 10.1107/S1600536811018198/lh5246Isup3.cml

Additional supplementary materials:  crystallographic information; 3D view; checkCIF report

## Figures and Tables

**Table 1:** Hydrogen-bond geometry (Å, °)

*D*—H⋯*A*	*D*—H	H⋯*A*	*D*⋯*A*	*D*—H⋯*A*
N1—H1*A*⋯O1	0.88	1.79	2.674 (1)	176
N3—H3*B*⋯O1^i^	0.88	2.03	2.835 (1)	151
N3—H3*C*⋯O2	0.88	2.08	2.902 (1)	155
C10—H10*A*⋯O3^ii^	0.95	2.46	3.1035 (14)	124
C11—H11*A*⋯O3^iii^	0.95	2.56	3.3629 (14)	143

Symmetry codes: (i) 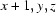; (ii) 
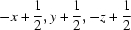
; (iii) 
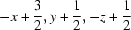
.

**Fig. 1.**
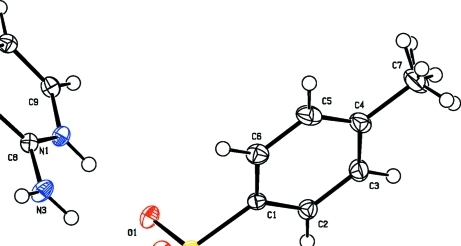
The molecular structure of the title compound with ellipsoids drawn at the 50% probabilty level.

**Fig. 2.**
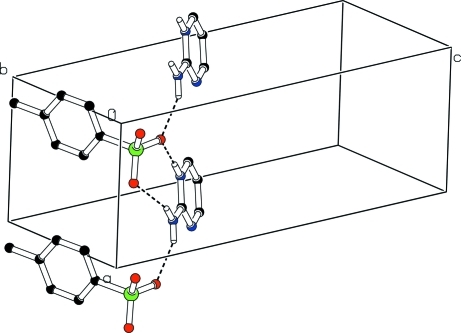
Part of a hydrogen-bonded (dashed lines ) chain along [100].

## supplementary crystallographic information

### Comment

The treatment of sulfonylchloride compounds with amines at room temperature leads to the corresponding sulfonamides (Tabatabaee *et al.*, 2010, 2011). The title compound was obtained as a side product from the reaction of 4-methylbenzenesulfonyl chloride and 2-amino-pyrimidine in CH_2_Cl_2_ under reflux conditions. The compound was formed due to the hydrolysis of 4-methylbenzenesulfonyl chloride to 4-methylbenzenesulfonic acid and an H atom being transferred to an imine nitrogen atom of 2-amino-pyrimidine molecule. The molecular structure of the title compound is shown in Fig. 1. The H atoms of the  methyl group are disordered over two sets of sites with equal occupancies. In the crystal, cations and anions are linked into one dimensional chains parallel to [100] (Fig. 2) by intermolecular N—H··· O hydrogen bonds and further stabilization is provided by weak C—H··· O hydrogen bonds. There is an intermolecular π···π stacking interaction involving pyrimidine and benzene rings with a centroid to centroid distance of 3.6957 (7)Å.

### Experimental

A solution of 2-amino-pyrimidine (0.095 g, 1 mmol) in CH_2_Cl_2_ (30 ml) was treated with 4-methylbenzenesulfonyl chloride (0.190 g, 1 mmol) and the pH of reaction mixture was adjusted to 8 with sodium carbonate solution (10%). The reaction mixture was refluxed. the solid crude was filtered. The clear filtrate solution was kept at 277K to give the colorless single crystals of the title compound.

### Refinement

All hydrogen atoms were visible in difference Fourier maps but were subsequently placed in calculated positions with C-H = 0.95-0.98Å and N-H = 0.88Å and refined in a riding-model approximation with *U*_iso_(H) = 1.2 *U*_eq_(C,N) or 1.5 *U*_eq_(C_methyl_).

### Crystal data

**Table:**

C_4_H_6_N_3_^+^·C_7_H_7_O_3_S^−^	*F*(000) = 560
*M**_r_* = 267.30	*D*_x_ = 1.419 Mg m^−^^3^
Monoclinic, *P*2_1_/*n*	Mo *K*α radiation, λ = 0.71073 Å
Hall symbol: -P 2yn	Cell parameters from 5926 reflections
*a* = 6.2567 (3) Å	θ = 2.7–34.6°
*b* = 13.3756 (6) Å	µ = 0.26 mm^−^^1^
*c* = 15.2512 (7) Å	*T* = 100 K
β = 101.335 (1)°	Prism, colourless
*V* = 1251.43 (10) Å^3^	0.50 × 0.36 × 0.32 mm
*Z* = 4	

### Data collection

**Table:**

Bruker SMART APEXII diffractometer	3160 reflections with *I* > 2σ(*I*)
Radiation source: fine-focus sealed tube	*R*_int_ = 0.021
graphite	θ_max_ = 30.0°, θ_min_ = 2.0°
ω scans	*h* = −8→8
12275 measured reflections	*k* = −18→18
3617 independent reflections	*l* = −21→21

### Refinement

**Table:**

Refinement on *F*^2^	Primary atom site location: structure-invariant direct methods
Least-squares matrix: full	Secondary atom site location: difference Fourier map
*R*[*F*^2^ > 2σ(*F*^2^)] = 0.032	Hydrogen site location: difference Fourier map
*wR*(*F*^2^) = 0.088	H-atom parameters constrained
*S* = 1.04	*w* = 1/[σ^2^(*F*_o_^2^) + (0.0496*P*)^2^ + 0.3689*P*] where *P* = (*F*_o_^2^ + 2*F*_c_^2^)/3
3617 reflections	(Δ/σ)_max_ = 0.001
163 parameters	Δρ_max_ = 0.45 e Å^−^^3^
0 restraints	Δρ_min_ = −0.38 e Å^−^^3^

### Special details

**Table:**

Geometry. All e.s.d.'s (except the e.s.d. in the dihedral angle between two l.s. planes) are estimated using the full covariance matrix. The cell e.s.d.'s are taken into account individually in the estimation of e.s.d.'s in distances, angles and torsion angles; correlations between e.s.d.'s in cell parameters are only used when they are defined by crystal symmetry. An approximate (isotropic) treatment of cell e.s.d.'s is used for estimating e.s.d.'s involving l.s. planes.
Refinement. Refinement of *F*^2^ against ALL reflections. The weighted *R*-factor *wR* and goodness of fit *S* are based on *F*^2^, conventional *R*-factors *R* are based on *F*, with *F* set to zero for negative *F*^2^. The threshold expression of *F*^2^ > σ(*F*^2^) is used only for calculating *R*-factors(gt) *etc*. and is not relevant to the choice of reflections for refinement. *R*-factors based on *F*^2^ are statistically about twice as large as those based on *F*, and *R*- factors based on ALL data will be even larger.

### Fractional atomic coordinates and isotropic or equivalent isotropic displacement parameters (Å^2^)

**Table:**

	*x*	*y*	*z*	*U*_iso_*/*U*_eq_	Occ. (<1)
S1	0.28924 (4)	0.172328 (19)	0.105904 (16)	0.01392 (8)	
O1	0.28421 (13)	0.21695 (6)	0.19437 (5)	0.01877 (17)	
O2	0.51293 (13)	0.15402 (6)	0.09590 (5)	0.01855 (16)	
O3	0.14528 (13)	0.08711 (6)	0.08830 (5)	0.01966 (17)	
C1	0.18525 (17)	0.26582 (8)	0.02687 (7)	0.01501 (19)	
C2	−0.03778 (18)	0.27159 (8)	−0.00789 (7)	0.0183 (2)	
H2A	−0.1349	0.2244	0.0096	0.022*	
C3	−0.1167 (2)	0.34700 (9)	−0.06827 (8)	0.0220 (2)	
H3A	−0.2687	0.3509	−0.0919	0.026*	
C4	0.0232 (2)	0.41735 (9)	−0.09488 (8)	0.0236 (2)	
C5	0.2456 (2)	0.41027 (9)	−0.05918 (8)	0.0254 (2)	
H5A	0.3429	0.4576	−0.0764	0.031*	
C6	0.3277 (2)	0.33500 (9)	0.00123 (8)	0.0213 (2)	
H6A	0.4797	0.3309	0.0248	0.026*	
C7	−0.0643 (3)	0.49831 (10)	−0.16093 (9)	0.0348 (3)	
H7A	−0.1992	0.4752	−0.1997	0.052*	0.50
H7B	0.0438	0.5140	−0.1975	0.052*	0.50
H7C	−0.0944	0.5584	−0.1286	0.052*	0.50
H7D	0.0326	0.5565	−0.1508	0.052*	0.50
H7E	−0.2103	0.5178	−0.1530	0.052*	0.50
H7F	−0.0721	0.4734	−0.2219	0.052*	0.50
N1	0.61962 (15)	0.31572 (7)	0.29233 (6)	0.01640 (18)	
H1A	0.5121	0.2804	0.2612	0.020*	
N2	0.99877 (15)	0.33985 (7)	0.33945 (6)	0.01665 (18)	
N3	0.86604 (16)	0.21369 (8)	0.23970 (7)	0.0210 (2)	
H3B	1.0008	0.1961	0.2382	0.025*	
H3C	0.7560	0.1808	0.2076	0.025*	
C8	0.82865 (17)	0.29000 (8)	0.29050 (7)	0.0154 (2)	
C9	0.57354 (18)	0.39454 (8)	0.34102 (7)	0.0177 (2)	
H9A	0.4264	0.4116	0.3415	0.021*	
C10	0.73953 (18)	0.44934 (8)	0.38933 (7)	0.0179 (2)	
H10A	0.7132	0.5064	0.4228	0.022*	
C11	0.95194 (17)	0.41679 (8)	0.38686 (7)	0.0170 (2)	
H11A	1.0701	0.4525	0.4217	0.020*	

### Atomic displacement parameters (Å^2^)

**Table:**

	*U*^11^	*U*^22^	*U*^33^	*U*^12^	*U*^13^	*U*^23^
S1	0.01196 (12)	0.01471 (13)	0.01493 (13)	−0.00058 (8)	0.00229 (9)	−0.00101 (8)
O1	0.0151 (4)	0.0257 (4)	0.0156 (3)	−0.0016 (3)	0.0034 (3)	−0.0040 (3)
O2	0.0135 (4)	0.0209 (4)	0.0214 (4)	0.0022 (3)	0.0038 (3)	−0.0016 (3)
O3	0.0186 (4)	0.0161 (4)	0.0234 (4)	−0.0036 (3)	0.0017 (3)	0.0011 (3)
C1	0.0159 (5)	0.0137 (4)	0.0158 (4)	0.0009 (4)	0.0039 (4)	−0.0015 (4)
C2	0.0171 (5)	0.0189 (5)	0.0186 (5)	0.0003 (4)	0.0028 (4)	−0.0008 (4)
C3	0.0225 (5)	0.0222 (5)	0.0200 (5)	0.0044 (4)	0.0006 (4)	−0.0002 (4)
C4	0.0361 (7)	0.0172 (5)	0.0175 (5)	0.0044 (5)	0.0056 (4)	0.0002 (4)
C5	0.0324 (6)	0.0185 (5)	0.0274 (6)	−0.0025 (5)	0.0107 (5)	0.0029 (4)
C6	0.0191 (5)	0.0203 (5)	0.0252 (5)	−0.0023 (4)	0.0063 (4)	0.0008 (4)
C7	0.0563 (9)	0.0225 (6)	0.0245 (6)	0.0088 (6)	0.0054 (6)	0.0064 (5)
N1	0.0124 (4)	0.0201 (4)	0.0166 (4)	−0.0024 (3)	0.0026 (3)	−0.0015 (3)
N2	0.0138 (4)	0.0189 (4)	0.0169 (4)	−0.0014 (3)	0.0021 (3)	−0.0005 (3)
N3	0.0145 (4)	0.0258 (5)	0.0231 (5)	−0.0018 (4)	0.0043 (3)	−0.0087 (4)
C8	0.0138 (5)	0.0182 (5)	0.0145 (4)	−0.0008 (4)	0.0035 (3)	0.0012 (4)
C9	0.0163 (5)	0.0193 (5)	0.0180 (5)	0.0028 (4)	0.0048 (4)	0.0016 (4)
C10	0.0191 (5)	0.0164 (5)	0.0186 (5)	0.0010 (4)	0.0044 (4)	0.0000 (4)
C11	0.0165 (5)	0.0169 (5)	0.0168 (5)	−0.0029 (4)	0.0013 (4)	0.0003 (4)

### Geometric parameters (Å, °)

**Table:**

S1—O3	1.4449 (8)	C7—H7C	0.9800
S1—O2	1.4580 (8)	C7—H7D	0.9800
S1—O1	1.4814 (8)	C7—H7E	0.9800
S1—C1	1.7697 (11)	C7—H7F	0.9800
C1—C6	1.3930 (15)	N1—C9	1.3529 (14)
C1—C2	1.3941 (15)	N1—C8	1.3579 (14)
C2—C3	1.3895 (16)	N1—H1A	0.8800
C2—H2A	0.9500	N2—C11	1.3233 (14)
C3—C4	1.3985 (18)	N2—C8	1.3500 (14)
C3—H3A	0.9500	N3—C8	1.3296 (14)
C4—C5	1.3946 (19)	N3—H3B	0.8800
C4—C7	1.5067 (17)	N3—H3C	0.8800
C5—C6	1.3929 (17)	C9—C10	1.3631 (15)
C5—H5A	0.9500	C9—H9A	0.9500
C6—H6A	0.9500	C10—C11	1.4060 (15)
C7—H7A	0.9800	C10—H10A	0.9500
C7—H7B	0.9800	C11—H11A	0.9500
			
O3—S1—O2	115.10 (5)	H7B—C7—H7D	56.3
O3—S1—O1	111.29 (5)	H7C—C7—H7D	56.3
O2—S1—O1	110.83 (5)	C4—C7—H7E	109.5
O3—S1—C1	107.38 (5)	H7A—C7—H7E	56.3
O2—S1—C1	106.22 (5)	H7B—C7—H7E	141.1
O1—S1—C1	105.36 (5)	H7C—C7—H7E	56.3
C6—C1—C2	120.29 (10)	H7D—C7—H7E	109.5
C6—C1—S1	119.39 (8)	C4—C7—H7F	109.5
C2—C1—S1	120.31 (8)	H7A—C7—H7F	56.3
C3—C2—C1	119.41 (11)	H7B—C7—H7F	56.3
C3—C2—H2A	120.3	H7C—C7—H7F	141.1
C1—C2—H2A	120.3	H7D—C7—H7F	109.5
C2—C3—C4	121.30 (11)	H7E—C7—H7F	109.5
C2—C3—H3A	119.4	C9—N1—C8	121.24 (9)
C4—C3—H3A	119.4	C9—N1—H1A	119.4
C5—C4—C3	118.33 (11)	C8—N1—H1A	119.4
C5—C4—C7	120.89 (12)	C11—N2—C8	116.81 (10)
C3—C4—C7	120.78 (12)	C8—N3—H3B	120.0
C6—C5—C4	121.14 (11)	C8—N3—H3C	120.0
C6—C5—H5A	119.4	H3B—N3—H3C	120.0
C4—C5—H5A	119.4	N3—C8—N2	119.44 (10)
C5—C6—C1	119.52 (11)	N3—C8—N1	119.13 (10)
C5—C6—H6A	120.2	N2—C8—N1	121.43 (10)
C1—C6—H6A	120.2	N1—C9—C10	119.59 (10)
C4—C7—H7A	109.5	N1—C9—H9A	120.2
C4—C7—H7B	109.5	C10—C9—H9A	120.2
H7A—C7—H7B	109.5	C9—C10—C11	116.28 (10)
C4—C7—H7C	109.5	C9—C10—H10A	121.9
H7A—C7—H7C	109.5	C11—C10—H10A	121.9
H7B—C7—H7C	109.5	N2—C11—C10	124.57 (10)
C4—C7—H7D	109.5	N2—C11—H11A	117.7
H7A—C7—H7D	141.1	C10—C11—H11A	117.7
			
O3—S1—C1—C6	−152.15 (9)	C7—C4—C5—C6	−179.36 (12)
O2—S1—C1—C6	−28.51 (10)	C4—C5—C6—C1	−0.34 (18)
O1—S1—C1—C6	89.14 (9)	C2—C1—C6—C5	0.24 (17)
O3—S1—C1—C2	29.29 (10)	S1—C1—C6—C5	−178.32 (9)
O2—S1—C1—C2	152.94 (9)	C11—N2—C8—N3	178.22 (10)
O1—S1—C1—C2	−89.42 (9)	C11—N2—C8—N1	−2.14 (15)
C6—C1—C2—C3	−0.06 (16)	C9—N1—C8—N3	−178.11 (10)
S1—C1—C2—C3	178.49 (9)	C9—N1—C8—N2	2.25 (16)
C1—C2—C3—C4	−0.03 (17)	C8—N1—C9—C10	−0.06 (16)
C2—C3—C4—C5	−0.07 (18)	N1—C9—C10—C11	−1.95 (15)
C2—C3—C4—C7	179.54 (11)	C8—N2—C11—C10	−0.04 (16)
C3—C4—C5—C6	0.25 (18)	C9—C10—C11—N2	2.07 (16)

### Hydrogen-bond geometry (Å, °)

**Table:**

*D*—H···*A*	*D*—H	H···*A*	*D*···*A*	*D*—H···*A*
N1—H1A···O1	0.88	1.79	2.674 (1)	176
N3—H3B···O1^i^	0.88	2.03	2.835 (1)	151
N3—H3C···O2	0.88	2.08	2.902 (1)	155
C10—H10A···O3^ii^	0.95	2.46	3.1035 (14)	124
C11—H11A···O3^iii^	0.95	2.56	3.3629 (14)	143

Symmetry codes: (i) *x*+1, *y*, *z*; (ii) −*x*+1/2, *y*+1/2, −*z*+1/2; (iii) −*x*+3/2, *y*+1/2, −*z*+1/2.
